# Supplementary material for: EnzML: multi-label prediction of enzyme classes using InterPro signatures
Source: BMC Bioinformatics. 2012 Apr 25;13:61. doi: 10.1186/1471-2105-13-61 (PMC3483700; doi:10.1186/1471-2105-13-61)
Supplement: Addtional file 5 — The Java code to format the data files, evaluate and predict. The file enzml_java_code.tar.gz contains the Java code used to format database data to ARFF and XML formats, to execute cross and train-test (jackknife) evaluations and to record evaluation results to database. More information is included in the readme.txt file and the Javadoc files. The code can be used with a MySQL database. To use a different database software, other JDBC drivers might be required. [file 1471-2105-13-61-S5.gz › java_code/enzml2011/doc/uk/ac/ed/inf/taweka/mulan/learn/TrainTestExperimenter.html]

TrainTestExperimenter


---


|  |  |  |
| --- | --- | --- |
| **Overview** \| **Package** \| **Class** \| **Use** \| **Tree** \| **Deprecated** \| **Index** \| **Help** | |  |
| **PREV CLASS**   **NEXT CLASS** | **FRAMES**    **NO FRAMES**     **All Classes** |  |
| SUMMARY: NESTED \| FIELD \| CONSTR \| METHOD | DETAIL: FIELD \| CONSTR \| METHOD |  |


---


## uk.ac.ed.inf.taweka.mulan.learn Class TrainTestExperimenter

```
java.lang.Object
  uk.ac.ed.inf.taweka.Initialised
      uk.ac.ed.inf.taweka.mulan.learn.MulanCrossExperimenter
          uk.ac.ed.inf.taweka.mulan.learn.TrainTestExperimenter
```

**All Implemented Interfaces:**: Initialisable

**Direct Known Subclasses:**: TrainTestExperimenterSerialized

---

``` public class TrainTestExperimenter extends MulanCrossExperimenter ```

Using Mulan (multi-label machine learning), this class initialises and
executes Train-Test evaluation (two arff records are needed, for the training
and testing files) (calling `MulanTrainTestEvaluator`). Initialisation
includes verifying input (train arff, test arff and train xml files) and
checking output locations (for saving evaluation statistics and predictions
to file and database).

**Version:**
:   29 Sep 2010

**Author:**
:   Luna De Ferrari luna.deferrari-at-ed.ac.uk

---

**Field Summary**

**Fields inherited from class uk.ac.ed.inf.taweka.Initialised**

`m_initialisationIsCorrect`


**Constructor Summary**

`TrainTestExperimenter(java.lang.String experimentPropsFile,
java.lang.String arffRecordDbProps,
int trainArffRecordId,
int testArffRecordId,
MulanLearner learner)`


| **Method Summary** | |
| --- | --- |
| `void` | `evaluateAndSave()` |
| `TrainTestEvaluator` | `getEvaluator()` |
| `MulanArffRecord` | `getTestArffRecord()` |

**Methods inherited from class uk.ac.ed.inf.taweka.mulan.learn.MulanCrossExperimenter**

`crossEvaluateAndSaveResults, evaluateAndSave, getExperimentDbManager, getLearner, getResultsSaver, getTrainArffRecord, main`

**Methods inherited from class uk.ac.ed.inf.taweka.Initialised**

`initialisationIsCorrect, setInitialisationIsCorrect`

**Methods inherited from class java.lang.Object**

`equals, getClass, hashCode, notify, notifyAll, toString, wait, wait, wait`

**Constructor Detail**

### TrainTestExperimenter

```
public TrainTestExperimenter(java.lang.String experimentPropsFile,
                             java.lang.String arffRecordDbProps,
                             int trainArffRecordId,
                             int testArffRecordId,
                             MulanLearner learner)
```


**Method Detail**

### evaluateAndSave

```
public void evaluateAndSave()
```

---


### getEvaluator

```
public TrainTestEvaluator getEvaluator()
```

:   **Overrides:**: `getEvaluator` in class `MulanCrossExperimenter`

---


### getTestArffRecord

```
public MulanArffRecord getTestArffRecord()
```


---


|  |  |  |
| --- | --- | --- |
| **Overview** \| **Package** \| **Class** \| **Use** \| **Tree** \| **Deprecated** \| **Index** \| **Help** | |  |
| **PREV CLASS**   **NEXT CLASS** | **FRAMES**    **NO FRAMES**     **All Classes** |  |
| SUMMARY: NESTED \| FIELD \| CONSTR \| METHOD | DETAIL: FIELD \| CONSTR \| METHOD |  |


---
